# The Genomics of Myelodysplastic Syndromes: Origins of Disease Evolution, Biological Pathways, and Prognostic Implications

**DOI:** 10.3390/cells9112512

**Published:** 2020-11-20

**Authors:** Hassan Awada, Bicky Thapa, Valeria Visconte

**Affiliations:** 1 Department of Translational Hematology and Oncology Research, Taussig Cancer Institute, Cleveland Clinic, Cleveland, OH 44106, USA; awadah@ccf.org; 2 Division of Hematology and Oncology, Medical College of Wisconsin, Milwaukee, WI 53226, USA; bithapa@mcw.edu

**Keywords:** MDS, mutations, deregulated expression

## Abstract

The molecular pathogenesis of myelodysplastic syndrome (MDS) is complex due to the high rate of genomic heterogeneity. Significant advances have been made in the last decade which elucidated the landscape of molecular alterations (cytogenetic abnormalities, gene mutations) in MDS. Seminal experimental studies have clarified the role of diverse gene mutations in the context of disease phenotypes, but the lack of faithful murine models and/or cell lines spontaneously carrying certain gene mutations have hampered the knowledge on how and why specific pathways are associated with MDS pathogenesis. Here, we summarize the genomics of MDS and provide an overview on the deregulation of pathways and the latest molecular targeted therapeutics.

## 1. Introduction

Myelodysplastic syndrome (MDS) is characterized by the dysregulation in hematopoiesis leading to uni-/multi-lineage dysplasia in the bone marrow, peripheral blood cytopenias, and increased risk of progression to acute myeloid leukemia (AML) [1,2,3]. Significant advances have been made in the last decade which elucidated the pathobiology of MDS; however, the molecular pathogenesis of MDS remains complex due to the high rate of genomic heterogeneity. The intricacy is possibly due to the interaction of genetic mutations in hematopoietic stem cells (HSCs), bone marrow milieu, and extrinsic factors e.g., dysregulated immunity and chemoradiation therapy [4,5,6]. The incidence of MDS is more common in elderly individuals, highlighting the increased risk of acquired mutations in HSCs and progenitor cells (HSPCs), which keep on accumulating with age and reach on average 5–10 mutations [7,8]. The recurrent genomic mutations affect diverse pathways such as RNA splicing, epigenetics, transcription, signaling, and metabolism [4,9]. Studies suggest that genomic aberrations in MDS are most commonly due to somatic mutations followed by chromosomal abnormalities and less often due to germline mutations [4,10,11]. In children, several rare genetic bone marrow failure syndromes predispose to an increased risk of MDS, such as Fanconi anemia, dyskeratosis congenita or telomeropathies, and Shwachman-diamond syndrome [12]. Besides, we have more evidence of increased risk of MDS with germline mutations affecting genes such as *ANKRD26*, *CEBPA*, *DDX41*, *ETV6*, *GATA2*, and *RUNX1* [4,13]. Therapy-related MDS (t-MDS) is another spectrum of MDS attributed to the widespread of radiation therapy and treatment with high dose chemotherapy (cyclophosphamide, melphalan, busulfan, and ifosfamide), and topoisomerase II inhibitors (anthracyclines) [14,15]. This MDS subtype is frequently associated with chromosomal abnormalities/complex karyotypes [16]. Broadly, the most common genes with recurrent somatic mutations in MDS include *ASXL1* (10–20%), *EZH2* (5–10%), *NRAS* (5–10%), *RUNX1* (10–15%), *SF3B1* (20–30%), *SRSF2* (10–15%), *STAG2* (5–10%), *TET2* (20–30%), *TP53* (10–12%), and *U2AF1* (5–12%) [4,9,11,13]. Given the high complexity of MDS, it is critical to understand the genomic landscape of patients for disease classification, risk prognostication, and response to therapies. Here, we aim to review the comprehensive genomic profiles and understand the pathobiology of MDS by giving an overview of the recent advances in the field.

## 2. Chromosomal Abnormalities

Chromosomal abnormalities are common in MDS and found in about 50–60% of patients with conventional karyotyping [11,17,18,19]. The majority of chromosomal abnormalities are associated with t-MDS. Besides, there are diverse mechanisms that explain the occurrence of cytogenetic abnormalities, which include chromosomal loss, gain, amplification, and balanced translocations. Furthermore, point mutations in genes essential in DNA-repair mechanisms and/or chromosomal segregation can likely lead to duplications and deletions. Moreover, DNA methylation contributes to chromosome stability. Indeed, cells with decreased DNA methylation are more prone to undergoing chromosomal rearrangements, loss, and gain [20]. The abnormal DNA methylation patterns are also demonstrated in different cytogenetic risk groups.

Additionally, single-nucleotide polymorphism microarrays and array comparative genomic hybridization are utilized to detect chromosomal abnormalities [21,22], while fluorescence in situ hybridization (FISH) is used to detect microdeletion [23]. Given the heterogeneity of MDS, the diagnosis of MDS is assessed by various morphologic tests on bone marrow smears and core biopsy (iron and Wright staining) and flow cytometric analysis to define the cellular immunophenotype.

In this section, we review the most common chromosomal abnormalities in MDS.

### 2.1. Chromosome 5q Deletion [del(5q)]

The deletion of the long arm of chromosome 5 (5q) is usually interstitial and represents the most common chromosomal abnormality found in MDS. The incidence of del(5q) in MDS patients ranges from 10–15% [24]. Patients with isolated 5q-syndrome (sole chromosomal abnormality) have a favorable prognosis with a relatively benign clinical course, a lower risk of transformation to leukemia, and a good response to immunomodulatory drugs e.g., lenalidomide [25]. On the contrary, isolated karyotypic abnormality of del(5q) in AML is associated with a poor prognosis [26]. Other subtypes of del(5q) are associated with complex karyotype, *TP53* mutations, or 17p loss [27]. These subtypes correlate with poor prognosis, increased risk of transformation to AML, and no response to lenalidomide [26,27]. Nevertheless, del(5q) is characterized by complex pathobiology in MDS due to gene deletion, which most frequently occurs in commonly deleted regions (CDRs) e.g., 5q31.1 and 5q32-33.3 regions [28]. Evidence suggests that haploinsufficiency of the genes located in the CDRs dictates different pathological manifestations in patients with del(5q). In fact, the gene expression analysis of cells derived from patients with the 5q- syndrome showed abnormal expression of genes involved in ribosome biogenesis [29]. For example, the dyserythropoiesis in 5q- syndrome is due to haploinsufficiency of the ribosomal protein S14 (RPS14) located at 5q31.2 region, which leads to the activation of p53 in erythroid progenitors [30]. Experimental studies demonstrated that the partial loss of function of RPS14 acts like 5q- syndrome in normal HSCs. Exogenous re-expression of RPS14 rescues the phenotype in cells of patients with 5q- syndrome [30]. Studies have also indicated that microRNAs might be involved in MDS pathogenesis by regulating HSCs. Loss of one copy of the microRNA miR-145 and miR146a might be a trigger for clonal advantage and specific morphologic features. For instance, megakaryocytic dysplasia and thrombocytosis are manifested due to dysregulation of microRNA (miRNA), which leads to depletion of miRNA-145 and miRNA-146a. The latter has been implicated in hematopoietic progenitors’ self-renewal and lineage commitment [31].

In terms of therapeutic benefits, lenalidomide is effective in cases with haploinsufficiency of *CSNK1A1* gene located on 5q32 region and encoding casein kinase 1 alpha 1 [32]. Other haploinsufficient genes in del(5q) include *APC*, *EGR1*, *NPM1*, and *HSPA9* [33,34,35].

### 2.2. Chromosome 7q Deletion [del(7q)], Monosomy 7 (-7)

Partial or total loss of chromosome 7 is observed in MDS. It is hypothesized that the pathogenesis of del(7q) or -7 is due to haploinsufficiency of genes at CDRs 7q22, 7q32-33, and 7p35-36 [36]. However, the exact pathogenesis is yet to be elucidated. Evidence suggests that genes involved in the pathogenesis of del(7q) include *CUX1*, *EZH2*, *MLL3*, *SAMD9*, *SAMD9L* [37,38,39,40]; however, the clinical phenotype of this abnormality cannot be attributable to only one gene. Recent studies have suggested that del(7q)/-7 is an early event in HSPCs. HSPCs might remove *SAMD9/9L* gain-of-function mutations via aneuploidy and acquire a competitive advantage [41]. Mixed-lineage leukemia 3 (*MLL3*) encodes for a protein containing a SET domain capable of methylating lysine 4 on histone H3, which is associated with active transcription. Studies have shown that *MLL3* controls the differentiation of HSPCs. Suppression of *MLL3* was insufficient to induce leukemia, but cooperation with loss of p53 can impair the differentiation of HSPCs and induce a phenotype that resembled MDS [39]. Del(7q) is present in approximately 50% of patients with t-MDS and about 10% of patients with de novo MDS [22,42,43]. MDS patients carrying del(7q) have a dismal prognosis [44].

### 2.3. Trisomy 8

About 5–10% of patients with MDS have chromosome aberrations, including trisomy 8 (+8) [45]. The pathogenesis of +8 is not entirely understood; however, cytogenetic abnormalities with +8 tend to appear late during MDS [46]. AML patients with +8 carry mutations in DNA methylation, RNA-splicing, and transcription factor genes. A high frequency of mutations has been found in the transcription factor *RUNX1* as well as in the chromatin modifier *ASXL1* [47]. Amplification of *c-MYC* proto-oncogene in patients with AML with +8 has been postulated to be a driver of clonal evolution. Studies were done to assess whether MYC expression proportionally increases with the gain of chromosome 8. The mean expression of MYC levels in AML patients with +8 was higher compared to that of patients with AML and normal karyotype [48]. Studies have also shown that the presence of autoimmune manifestations is associated with trisomy 8, and in these cases, patients show a good response to immunosuppressive therapies [49,50].

### 2.4. Chromosome 20 q Deletion [del(20q)]

This abnormality is observed in approximately 2% of patients with MDS [22]. Sole del(20q) is present in the early stages of MDS and has been associated with a favorable prognosis [51,52]. Isolated del(20q) is commonly associated with thrombocytopenia, higher reticulocytes count, and lower numbers of blasts in the bone marrow [52]. When present in advanced MDS stages, del(20q) is often associated with particular gene mutations such as *ASXL1* and *U2AF1*. Several investigations were made to identify genes playing a pathogenetic role in the del(20q) CDR, but so far, no unique driver gene has been discovered. The expression level of BCAS4, ADA, and YWHAB genes was found to be significantly lower in MDS patients without del(20q) as compared to controls [53]. More recently, gene expression analysis identified the Hippo kinase MST1 STK4 which is down-regulated below haploinsufficient levels in MDS and MPN. Mice with inactivated Hippo kinase manifest splenomegaly, thrombocytopenia, megakaryocytic dysplasia, and a chronic granulocytosis, which are all frequently observed in patients with del(20q) [54].

### 2.5. Chromosome 17Abnormalities [17p Deletions, Isochromosome 17q]

In MDS patients, the 17p deletion is frequently associated with *TP53* mutations and unfavorable prognosis [55,56]. 17p deletion is classified under the intermediate-risk category. It occurs in about 1% of MDS cases as a sole abnormality. Besides, patients with isochromosome 17q are characterized by severe anemia, high leukocytes with neutrophils expressing the pseudo-Pelger–Huët anomaly, and hyperplastic bone marrow. It is controversial whether cases with isochromosome 17q also carry *TP53* mutations. Mutations in *TP53* are reviewed in Section 3.4 and Section 4.4.

*-Y, -X:* Losses of sex chromosomes can also be associated with hematological malignancies. Isolated loss of the Y chromosome is frequently encountered as an age-related phenomenon in MDS and is associated with a very good prognosis [57]. On the other hand, isolated loss of the X chromosome is rare in MDS and holds an intermediate prognosis [58,59].

### 2.6. Complex Karyotypes

Complex karyotype is common in MDS and includes at least three or more cytogenetic abnormalities [60]. The presence of associated gene mutations can often occur in combination with complex karyotypes as the case of *TP53*. *TP53* mutations and complex karyotype confer poor prognosis [61,62].

### 2.7. Rare Chromosomal Abnormalities

MDS is highly cytogenetically unstable. Several rare abnormalities can be also detected including del(9q), del(11q), del(12p) or t(12p), del(13q), der(7)t(1;7)(q10;p10), inv(3)(q21q26.2), t(3:3) trisomy 11, 13, 14/14q, 21, 11q23/MLL translocations, t(6:9)(p23;q34), t(2;11)(p21;q23), t(11;16)(q23;p13.3), 1(1;3)(p36.3;q21), t(2;11)(p21;q23), t(3;21)(q26.2;q22.1), idic(X)(q13), t(17p) (unbalanced translocations), or i(17q) (e.g., loss of 17p).

## 3. Somatic Mutations

The phenotypic heterogeneity of MDS has been recently made easier to understand by the revolutionary molecular advances provided via the advent of DNA sequencing technologies, which crucially unraveled fascinating pathogenetic mechanisms underlying disease evolution, development, and progression [63,64]. It also contributed to the identification of the implications of invariant associations of recurrent somatic gene mutations involving peculiar molecular pathways and their clonal successions, on the clinical phenotype, prognosis, and therapeutic response in MDS [65]. Molecular mutations have been associated with distinct clinical phenotypes but more importantly, differences in molecular profiles have been seen at diverse disease stages of MDS. The different frequencies of mutations are possibly explained by the time of mutational acquisition. Mutations in transcription factors (*CEBPA*, GATA2, *RUNX1*) and activating signaling pathways (*FLT3*, NRAS/RAS) are commonly found in secondary AML. Mutations in the DNA methylation pathway (e.g., *TET2*) or the splicing factor gene, *SF3B1* are present at the MDS stage suggesting that they represent early disease lesions. A mutation that is mostly associated with low risk MDS (specifically the ones with RS) affects *SF3B1* gene. *TP53* mutations are another example of mutations associated with lowrisk MDS with del(5q). On the contrary, the presence of mutations in splicing factor genes (e.g, *SRSF2*, *U2AF1*) in addition to *EZH2* and *STAG2* is more indicative of high risk vs. low risk MDS. Given the fact that MDS patients carry a median of 9 somatic mutations with a lower number of mutations among low risk patients, it is conceivable that an appreciable number of genes in different pathways are susceptible to mutations [11]. Here, we review mutations found to affect components of the RNA-splicing machinery [66,67,68,69,70], DNA methylation [71,72,73,74,75,76], histone modifications [77,78,79,80,81], transcription [82,83,84], signal transduction [85,86,87], and cohesion complex [88,89]. Collectively, these pathways contribute to MDS pathogenesis.

### 3.1. RNA-Splicing Machinery (SF3B1, SRSF2, U2AF1, ZRSR2)

The splicing factor 3b, subunit 1 (*SF3B1*) gene encodes a core component of the U2 nuclear ribonucleoprotein with the function of recognizing the 3′ splice site at intron-exon junctions. *SF3B1* gene is often a target for somatic mutations preferentially occurring in 4 consecutive HEAT (Huntington elongation factor 3 protein phosphatase 2A and the yeast PI3-kinase TOR1) domains of the C-terminal region, with the lysine to glutamic acid substitution at codon 700 (K700E) reported in the majority of cases with MDS and ringed sideroblasts (MDS-RS), a subtype of MDS identified as low-risk. Other common hotspot mutations involve the conserved amino acids 622, 625, 662, and 666. A small proportion of individuals with clonal hematopoiesis of indeterminate potential (CHIP), who are at risk for developing hematologic malignancies including MDS, have been found to be carriers of *SF3B1* mutations.

The U2-complex auxiliary factor 1 gene (*U2AF1*) encodes a 35-kDa protein of the U2-spliceosome responsible for the recognition of the terminal 3′ AG dinucleotide in pre-messenger RNA introns. The protein has 4 major domains, including two zinc finger regions, a serine-arginine (SR) domain, and an U2AF-homology domain. The U2AF1 unit forms a complex by heterodimerization with the 65-Kda protein called U2AF2 in order to bind at the 3′ end of introns and degrade the polypyrimidine tract. Mutations in *U2AF1* at codon S34 and Q157 are found in about 11% of patients with MDS and 4% of patients with AML. These hits are associated with worse survival outcomes and increased risk of AML transformation. Differences in S34 and Q157 have been investigated using in vitro models. It has been reported that S34 induces isoform changes in several mRNAs, including H2AFY and STRAP. These genes represent key downstream targets of S34F in myeloid cell lineages [90].

The serine and arginine-rich splicing factor 2 (*SRSF2*) gene is located on chromosome 17q25.2 and encodes a member of the SR-rich family of pre-mRNA splicing components. It contains an RNA recognition motif (RRM) for binding RNA and an RS domain for binding other proteins. The RS domain is enriched in SR residues, hence, facilitating the interaction between SR and diverse splicing factors. *SRSF2* mutations occur almost exclusively at the amino acid proline 95 and alter the binding affinity of the RRM motif. *SRSF2* mutations have been found in 28–47% of patients with CMML, about 14% of patients with MDS and have been associated with increased age, higher hemoglobin count, and normal cytogenetics. SRSF2 acts by direct targets and by alternative splicing of other splicing factors. In vitro studies demonstrated that the proteins targeted by *SRSF2*^P95H^ are mostly involved in RNA-processing and include members of the hnRNP and SR families of proteins. HITS-CLIP data showed an overrepresentation of HNRNP proteins (HNRNPA2B1, HNRNPH1, HNRNPM, HNRNPH3). Some of these proteins facilitate primary miRNA transcripts [91].

The zinc finger CCCH-type, RNA binding motif and serine/arginine-rich 2 (*ZRSR2*) is a gene located on chromosome Xp22.2 that encodes an SR-rich protein and is responsible for the recognition of the 3′ splice acceptor site the gene. *ZRSR2* is mutated in about 5% of patients with MDS. Mutations (out-of-frame insertions and deletions, nonsense, missense, splice site) occur mainly in male patients. The nature of the mutations resembles a loss-of-function profile.

### 3.2. DNA Methylation (DNMT3A, TET2)

The DNA (cytosine-5)-methyltransferase 3A encoded by the *DNMT3A* gene is involved in DNA methylation by transferring methyl groups to specific CpG structures in DNA. Although it is more commonly mutated in AML, *DNMT3A* mutations occur in about 11–13% of MDS and can be found in all MDS subtypes [92]. The most common mutation involves R882 amino acid residue. *DNMT3A* hits have shown adverse outcomes in MDS as *DNMT3A* mutant MDS patients had worse overall survival and increased progression to AML [75].

The tet methylcytosine dioxygenase 2 (*TET2*) gene plays a key role in inducing self-renewal and clonal expansion in HSC through demethylation which occurs by converting the methyl group at the 5-position of cytosine DNA 5-methylcytosine (5mc) to 5-hydroxy-methylcytosine (5hmc). *TET2* loss-of-function mutations lead to an impairment of DNA demethylation. *TET2* mutations occur in 20–25% of MDS patients and present with multiple types of pathogenetic variants encompassing the entire gene. While some studies showed no impact on overall survival [83,93], others displayed a favorable prognosis [94], although more recent studies revealed a shortened survival after allogeneic stem cell transplantation [95]. Interestingly, *TET2*-mutant MDS patients were more likely to respond to hypomethylating agents in clinical studies [96].

### 3.3. Histone Modification (ASXL1, EZH2, BCOR/BCORL1)

The additional sex combs like 1 (*ASXL1*) is an epigenetic modulator acting through post-translational histone modification. It contributes to the activation and suppression of *HOX* genes by regulating the polycomb group and trithorax group of proteins. *ASXL1* mutations occur in 15–24% of MDS patients with nonsense and frameshift variants affecting p.E635fs and p.G646fs. *ASXL1* mutations have been associated with worse prognosis and unfavorable survival outcomes in MDS as well as all other myeloid neoplasms [77].

The loss of function of the enhancer of zeste 2 polycomb repressive complex 2 subunit (*EZH2*) occurs in 7/7q- chromosome aberrations contributes to overexpression of the *HOX* gene clusters in MDS through epigenetic modifications [97]. Mutations mainly involve the SET-domain (p.R690) and occur in 5–8% of MDS. Overall, *EZH2* mutations are associated with poor outcomes [79,80,83].

The BCL6 corepressor and like 1 (*BCOR/BCORL1*) are components of the polycomb complex. In addition to being reported in aplastic anemia, paroxysmal nocturnal hemoglobinuria, and large granular lymphocytic leukemia [98,99,100], *BCOR/BCORL1* mutations in MDS cases have been described in 5% of the cases. The overall impact is controversial, likely due to the paucity of information. For instance, some studies showed unfavorable outcomes in MDS mutant *BCOR/BCORL1* [64,101,102], while another revealed a neutral impact even after adjustment to age and IPSS-R risk score [81].

### 3.4. DNA Transcription (RUNX1, TP53)

The RUNX family transcription factor 1 (*RUNX1*) mutations are frequently seen in 10–20% of MDS, especially higher-risk subtypes (e.g., MDS with multilineage dysplasia and MDS with excess blasts), and mainly occur as subclonal events [64]. The RUNX1 protein regulates the differentiation of HSPCs and other genes involved in hematopoiesis. As a result, *RUNX1* loss-of-function mutations play a crucial role in disease progression, as displayed by the acquisition of the mutations during leukemic transformation [103,104]. In some studies, *RUNX1* mutations were associated with severe thrombocytopenia and very poor outcomes [83,85].

The tumor protein P53 (*TP53*) gene is located on 17p13.11 and play a major role in regulating cell cycle arrest, cell senescence, apoptosis, and differentiation. *TP53* mutations are loss-of-function and seen in ~10% of MDS with frequencies of mutations increasing to about 50% in MDS patients with complex karyotype. *TP53* mutations are frequently associated with higher-risk MDS and t-MDS and carry a dismal prognosis. Remarkably, *TP53* mutations have the worst prognosis in MDS, including in patients who underwent HSC transplantation [82] and are linked to an increased risk of leukemic transformation [27]. A recent study from the International Working Group for Prognosis of MDS analyzed a large cohort (*n* = 3324) of de novo MDS and related myeloid neoplasms [105]. A comprehensive collection of cytogenetic data (G-banding analysis) and sequencing data (genome-wide copy number probes and capture-based NGS) were employed to resolve the clinical implications of *TP53* mutations and their allelic status in MDS. More importantly, the study highlighted the importance of considering *TP53* allelic status for diagnostic purposes and to guide clinical decisions. Differences in *TP53* allelic status were found between low and high-risk patients. Monoallelic *TP53* patients were commonly seen in lower risk MDS. The median OS was also found to be different according to allelic status in AML patients. AML patients carrying multi-hit state *TP53* had shorter OS compared to patients with monoallelic *TP53* mutations. Moreover, patients with monoallelic *TP53* status and variant allele frequency (VAF) >22% had an increased risk of death compared to wild type patients while patients with multi-hit state had poorer outcomes independently of VAF. The type of *TP53* mutations (missense, truncating, or hotspot) was also accounted in this study. In monoallelic status, the presence of mutations at hotspots R175 and R248 correlated with increased risk of death.

### 3.5. Signal Transduction (KRAS, NRAS, PTPN11)

The Kirsten rat sarcoma viral oncogene homolog (*KRAS*) and the neuroblastoma RAS viral oncogene homolog (*NRAS*) mutations have been reported in <5% of MDS patients. Mutations are missense and mainly affect positions D12, D13, and D61 hotspots. They often occur during MDS transformation to AML and have been associated with shortened survival in MDS [85]. The protein tyrosine phosphatase, non-receptor type 11 (*PTPN11*), is part of the RAS family pathway and rarely mutated in adult MDS (<1%) [106]. The missense mutations include N-SH2 and PTP domains and have been associated with juvenile myelomonocytic leukemia (JMML) and childhood MDS/AML [86,106]. Additionally, it was found that *PTPN11* mutations could be acquired during the progression of MDS or JMML [107].

### 3.6. Cohesion Complex (SMC3, SMC1A, RAD21, STAG2)

**Figure 1:**
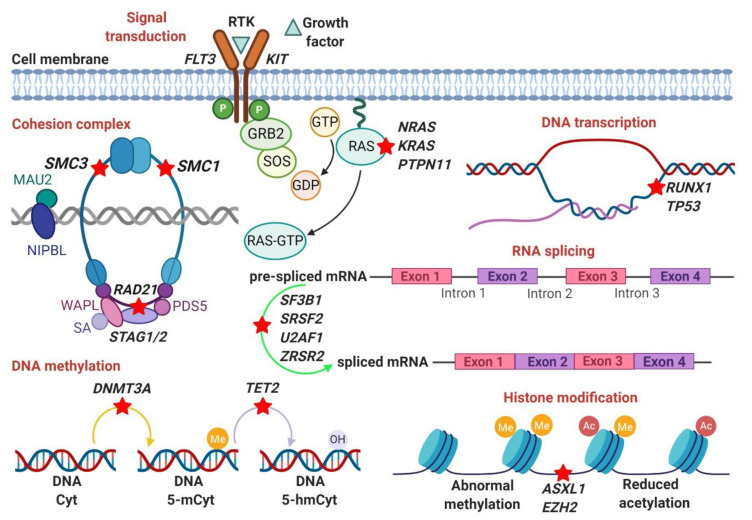
The altered genetic pathways in MDS. The figures illustrate the main genetic pathways involved in the pathogenesis of MDS including: Signal transduction (*KIT*, *FLT3*, *RTK*), Cohesion complex (*RAD21, SMC1/3, STAG1/2*), DNA transcription (*TP53, RUNX1*), RNA-splicing (*SF3B1*, *SRSF2*, *U2AF1*, *ZRSR2*), DNA methylation (*DNMT3A*, *TET2*), Histone modification (*ASXL1*, *EZH2*).

The cohesion complex is known to align and stabilize replicated chromosomes prior to cell division. Nonsense and frameshift mutations occur in four core subunits of the cohesin complex (*SMC3*, *SMC1A*, *RAD21*, *STAG2*), which serve as transcriptional coactivators. They are often heterozygous and result in haploinsufficiency or dominant-negative effects. As a whole, the mutational frequency is about 12–20% in AML and MDS with a particular prevalence in high-risk MDS and secondary AML. *STAG2* is the most mutated core subunits in AML [89]. Studies have demonstrated that *STAG2* mutations are very early events occurring in naïve stem cells and affecting dominant clones [108]. Figure 1 illustrates the main altered genetic pathways in MDS as discussed in this section.

## 4. Germline Mutations

Familial presentation of MDS due to germline variants are still mostly unexplored; however, NGS techniques are playing a pivotal role in understanding the pathobiology of these mutations. More recent insights have been generated for putative genes responsible for the inheritance of MDS/AML [109]. For instance, a study reported that about 45% of MDS/AML families had a mutation in genes including *RUNX1*, *TERT*, *ANKRD26*, *CEBPA*, *DDX41*, *ETV6*, *GATA2*, *TERC,* and *TP53* [109]. These gene variants had a significant association with the disease evolution in MDS/AML families. In the same study, the authors also found a moderate level of evidence to suggest gene-disease association in 5% of the families with a variant of known genes such as *CHEK2*, *ACD*, *RTEL1*, *SAMD9/9L*, *SRP72* [109]. Germline mutations of genes associated with MDS/AML include *CEBPA*, *DDX41*, *ETV6*, *GATA2*, and *RUNX1*. These genes show an autosomal dominant pattern of inheritance.

### 4.1. RUNX1

Individuals with a germline point mutation of *RUNX1* are at increased risk for developing AML/MDS at advanced age [110]. *RUNX1* is the transcription factor gene encoding the DNA binding alpha subunit of the core binding transcription factor (CBF). The heterodimeric CBF is the crucial regulator of the normal differentiation of HSCs [111].

### 4.2. GATA2

MDS associated with a germline mutation in *GATA2* is rare. *GATA2* gene encodes a transcription factor that regulates early HSC [112]. MDS patients with *GATA2* mutations are characterized by ineffective hematopoiesis, dysplasia, and high risk of progression to AML. The exact mechanism of the development of MDS in *GATA2* deficiency is not yet precise. However, subsets of patients with *GATA2* germline mutations are also found to have acquired coexisting *ASXL1* mutations, which could possibly explain the interaction at the molecular level leading to the pathogenesis of MDS [113].

### 4.3. ETV6

*ETV6* gene is located at 12p13.2 and encodes for a transcription factor, which is the DNA binding protein ETV6. It is present in <5% of the case of MDS [85]. The mutation of the *ETV6* gene can be either somatic or germline. Germline missense mutations of *ETV6* have been found in patients with MDS and AML [114]. In general, germline *ETV6* mutations lead to inherited thrombocytopenia and are associated with increased risk of hematologic malignancies such as MDS and acute leukemia [115].

### 4.4. TP53

TP53 mutations are frequently associated with a complex karyotype (described in Section 2.4), which predisposes to disease progression with poor prognosis [62]. Moreover, *TP53* mutations are commonly seen in patients with t-MDS or AML.

### 4.5. DDX41

The germline DEAD-box helicase 41 (DDX41) gene encode an RNA helicase protein. Evidence suggests that germline mutation of *DDX41* gene is common in patients with MDS/AML and is associated with favorable outcome [116,117].

### 4.6. CEBPA

CCAAT enhancer-binding protein alpha/CEBPA gene is located at 19q13.11, which encodes a transcription factor and identifies the CAAT motif in the promoters of target genes. Mutations of the *CEBPA* gene with aberrant methylation are commonly observed in AML and portend favorable prognosis [118]. However, in other studies, CEBPA methylation was usually found without holding any prognostic value [119].

### 4.7. Genetic Predisposition

The genetic predisposition to MDS differs between children and young adults compared to older adults, as the former age group tends to be affected by germ-line mutation while the disease in the latter group is more commonly due to age-related somatic mutations [120]. Several genetic mutations and inherited disorders have specifically contributed to the development of MDS in the 1st age group, including: the DNA repair FA genesset of Fanconi anemia, the *GATA1*/*RPS19* genes precipitating Diamond Blackfan anemia, *TP53* of Li-Fraumeni, *SBDS* gene in Shwachman-Diamond syndrome, *SRP72* gene of the signal recognition particle complex, and the severe congenital neutropenia-inducing *ELANE* gene [120]. As for the older adults group, somatic mutations of the *TET2* and *DNMT3A* genes and germline mutation of *DDX41* appear to be the most common predispositions that are specific to this group [4]. Moreover, MDS due to germline telomere biology disorders, *SAMD9*/*SAMD9* mutations, *GATA2* mutation and familial platelet disorders secondary to mutations in *RUNX1*, *ETV6*, or *ANKRD26* may present across all ages [120].

## 5. Clonal Hematopoiesis of Indeterminate Potential (CHIP)

Heterogeneous clonality and cytopenias are hallmark features of MDS; however, in certain individuals, the unexplained cytopenias or clonality do not fulfill the criteria of the World Health Organization for hematologic malignancy. NGS technologies are instrumental in detecting the clonality in hematologic malignancies. The diverse somatic mutations in MDS is itself a challenge to draw meaningful clinical implications. Interestingly, the somatic mutations observed in MDS are also found in individuals without any myeloid malignancies along with normal peripheral blood count. In addition, acquired mutations in MDS-associated genes are also common in unexplained cytopenia without hematologic malignancies [121,122]. Therefore, it is prudent to recognize clonal hematopoiesis (CH) in healthy elderly individuals to differentiate from MDS and understand its clinical relevance for further monitoring.

CH is characterized by one or more acquired mutations in HSCs. Individuals with CH without cytopenia, dysplasia, and any hematological malignancies are referred to as CHIP [121]. Other related CH with unexplained cytopenia without any evidence of underlying hematologic malignancies include idiopathic cytopenia of undetermined significance (ICUS) and clonal cytopenia of undetermined significance (CCUS).

The criteria for the diagnosis of CHIP includes [123]: (a) the occurrence of acquired mutations in a leukemia-associated gene at variant allele frequency ≥2%; (b) absence of persistent peripheral blood cytopenia or normal peripheral blood counts; c) lack of evidence suggestive of MDS and other myeloid malignancies. CHIP is a premalignant condition with a 0.5–1% risk of progression to a hematologic malignancy [123]. In terms of clinicopathological diagnosis, individuals with CHIP are considered to have MDS if there is evidence of sustained cytopenia for ≥4 months with dysplasia [124]. The most common leukemic driver genes include *ASXL1*, *DNMT3A*, *JAK2*, *TET2*, and *TP53* [123,125,126,127,128]. Other rare mutations in CHIP are *SRSF2*, *SF3B1*, *CBL*, *IDH1/2*, *BCOR/L1*, *U2AF1*, *CREBBP*, *GNB1*, *CUX1*, *MLL2*, *SETD2*, *SETDB1*, *PPM1D*, and *GNAS* (Table 1). The prevalence of these somatic mutations increases with age. CHIP is also associated with an increased risk of cardiovascular disease and overall mortality [121,129]. Evidence also suggests that CHIP may affect the outcome of bone marrow transplant patients. Although older allogeneic bone marrow transplant patients who received CHIP-positive donors had survival outcomes similar to donors without CHIP [130], however, younger recipients with CHIP-positive donors may have a potential risk of increased chronic graft vs. host disease, albeit similar survival outcomes [130]. On the other hand, CHIP is associated with poor survival and increased risk of therapy-related myeloid neoplasms in patients with lymphoma who underwent autologous stem cell transplantation (ASCT) [131].

## 6. Deregulation of Pathways in MDS

Deregulation of cellular pathways in MDS cells, specifically the ones that lead to clonal progression, has been intensively studied, although the lack of cellular models has hampered the clarification of unique signatures. The comparison of the gene expression profile of CD34^+^ cells of a large number of patients with MDS (*n* = 183) with that of CD34^+^ cells of normal individuals showed that half of the MDS cells expressed a signature of 35 genes which were deregulated. Levels of genes clustering in the interferon-stimulated family and signaling pathway, thrombopoietin receptor (TPOR), STAT1, and SOS1 were significantly up-regulated, while the most downregulated genes included the Wnt/β-catenin signaling pathway, B-cell receptor signaling, IL-4 signaling, and chemokine signaling such as CXCR4 which was down-regulated in almost half of patients with MDS-RS. Haploinsufficient expression was also found in two tumor suppressor genes, PLK4 and CHK1. Some pathways, such as Wnt/β-catenin signaling, cell cycle regulation, and integrins were explicitly down-regulated in del(5q) MDS as SAPK/JNK, NF-kB, PI3K/AKT were in -7/del(7q) MDS [132]. More recently, gene expression-based classification was applied to RNA-sequencing data derived from CD34^+^ cells of 100 MDS patients. The analysis identified two subcellular populations characterized by increased expression of genes related to erythroid/megakaryocytic lineages and genes related to immature progenitor cells. These two subtypes were also associated with clinical outcomes [133].

Several genes involved in the iron pathway have been identified to play a role in the pathogenesis of MDS. SLC25A37 (Mitoferrin-1), an iron importer localized in the inner membrane of the mitochondria, was found up-regulated in patients with MDS carrying *SF3B1* mutations [134]. Similarly, SLC25A38 was also found up-regulated [135]. ABCB7, a key transporter of clusters of iron and sulfur atoms (Fe-S clusters) outside the mitochondria, was found aberrantly spliced in *SF3B1* mutant MDS cells explaining its lower expression compared to cells of normal individuals [136]. High expression of heme biosynthesis-related genes (e.g., *ALAS2* and *FECH*) were found down-regulated. Novel technologies such as RNA-sequencing were recently used to identify changes in transcriptome either in total mRNA, isoforms, or low expressed transcripts. Several studies have been conducted to identify differences in downstream pathways, specifically for genes of the spliceosomal complex (*SF3B1*, *SRSF2*, *U2AF1*). All these studies showed convergent functions in the 3′ splice site (SS). Recent studies revealed that splicing factor mutations could lead to the accumulation of R-loops (RNA-DNA hybrids with a displaced single-stranded DNA). Particularly, mutations in two spliceosomal genes (*U2AF1*, *SRSF2*) increase R-loops’ formation in leukemia cells, causing DNA damage and replication stress. A recent study showed an increase in R-loops in an induced pluripotent stem cell clone harboring *SF3B1*^K700E^ mutation compared to a clone lacking *SF3B1* mutations from an MDS patient [137]. Abnormal splicing events associated with mutations in *SF3B1*, *SRSF2*, and *U2AF1* mutations include alternative 3′SS, retained intron (RI), and skipped exon (SE). The 3′SS events can result in a frameshift mutation leading to downregulation via non-mediated decay (NMD). RI events can lead to NMD and extend the half-life of mRNAs. Finally, SE events result in alternative isoform production. Events like RI and the use of alternative ’3SSs in cells with *SF3B1* mutations were identified for a gene called *ERCC3*, a transcriptional factor involved, and R-loop-mediated DNA damage. A group of 5 genes (*LST1*, *LUC7L*, *MRRF*, *ORMDL1*, *SUGP2*) were common in MDS cells with *SF3B1*, *SRSF2*, and *U2AF1*. Aberrant splicing also affected some genes mutated in MDS, for instance, *STAG2* in *SF3B1* and *SRSF2* mutant cases and *EZH2* and *BCOR* in *SRSF2* and *U2AF1* mutant cases [138]. These genes are also part of crucial pathways involved in DNA-transcription, epigenetic regulation, and signal transduction. More recently, splicing factor mutations were found to lead to the activation of inflammation and innate immunity pathways (e.g., NF-kB) or indirectly by deregulating histone acetylase (e.g., sirtuins). In fact, sirtuin 1 (SIRT1) was found to be involved in HSPC maintenance. *U2AF1* mutant patients had IRAK4, a serine/threonine that activates NF-kB in the Toll-like receptor and T-cell receptor signaling pathways, that is aberrantly spliced. This abnormal splicing produces a longer isoform of IKAK4 (IRAK4-L) due to the retention of intron 4. The importance of this finding resides in the importance of targeting IRAK4 to reduce the leukemogenic potential. In fact, the inhibition of IRAK4 in MDS xenografts reduced MDS engraftment. Recently, a greater interest has grown to study molecular mechanisms deregulating signaling via phosphoinositides due to their involvement in the formation of a second messenger implicated in cell–cell communications and signaling. PI-PLCβ1 and other inositide-kinases (PKCs, PI3K/Akt) are deregulated in MDS [139]. Mutations in genes related to the inositide signaling pathways (AKT3, PI3KCD, PLCG2) were associated with a low response to the hypomethylating agents and lenalidomide. The authors nicely described the dynamics of the acquisition of the three mutations (*PIK3CD*^D133E^, *AKTD*^280G^, *PLCG2*^Q548R^) while patients with high risk MDS slowly lost response to treatment [140].

Splicing factors have been also associated with increased formation of R-loops, DNA/RNA hybrid structures which are implicated in the maintenance of genomic instability. CD34^+^ cells of MDS patients with splicing factor mutations have abnormal splicing of DNA-damage genes (*ATR*, *SETX*) regulating R-loops formation [141]. A connection between methylation and splicing factors has also been investigated, suggesting that methylation might be a mechanism of silencing splicing factors. The promoter DNA hypermethylation was studied in splicing factors including, *SF3B1*, *SRSF2*, *U2AF1*, *ZRSR2*. With the exception of the hypermethylation of *ZRSR2* promoter that was detected in 4% of the cases without an associated decrease in mRNA expression levels, other splicing factors did not seem to be affected by abnormalities in methylation [142].

More recently, inhibitors of the protein arginine methyltransferases (PRMTs) have been shown to be associated with perturbation of RNA-splicing. PRMTs catalyze the methyl transfer to the arginine residues of protein substrates including splicing factors and it has been proposed that MDS cells carrying splicing factor mutations may have a different sensitivity to PRMTs inhibitors [143].

## 7. Prognostic Implications of Chromosomal and Mutational Alterations in MDS

Patients with MDS, despite their molecular and cytogenetic complexity, usually have vague clinical presentations such as fatigue, bleeding, and increased risk of infections. Clinical features can overlap with other illnesses and differential diagnosis could include nutritional deficiencies, alcoholism, liver diseases, toxins, autoimmune diseases, human immunodeficiency virus (HIV) infection, and other primary hematologic malignancies. Clinicians must rule out common disease conditions prior to the diagnosis of MDS. Bone marrow biopsy is usually supportive in making a definitive diagnosis of MDS. Notably, 10–20% of patients with MDS have a clinical presentation with autoimmune manifestation such as inflammatory arthritis, vasculitis, and connective tissue diseases [144]. Immune dysregulation has been implicated in the pathogenesis of autoimmune disease, and these patients respond to immune-suppressive agents such as corticosteroids [145,146]. Rare dermatologic manifestation includes neutrophilic dermatosis or Sweet’s syndrome [147], which is associated with an imminent transformation to AML. Neutrophilic dermatosis responds to therapy with dapsone or corticosteroids [148].

The identification of chromosomal abnormalities is clinically essential for prognostication and therapeutic implications. Isolated del(5q) is the only cytogenetic abnormality that is identified as a specific subtype in the World Health Organization classification of MDS [149]. Cytogenetics have proven to be independent predictors for outcomes and play a vital role in determining prognosis in MDS. The revised International Prognostic Scoring System (IPSS-R) is a widely used model for prognostication of chromosomal abnormalities [150]. The cytogenetic scoring system in IPSS-R is classified into 5 prognostic subgroups (very good, good, intermediate, poor, and very poor) based on the overall survival and risk of transformation to AML [59,150]. Isolated loss of Y chromosome and del(11q) are classified as very good prognostic subgroups. Del(5q), del(12p), and del(20q) are associated with good prognosis whereas del(7q), +8, +19 belong to intermediate prognostic subgroups. The presence of del(7q) and complex karyotype with 3 abnormalities are associated with poor prognosis. On the other hand, complex karyotype with greater than 3 abnormalities is classified as a very poor prognostic subgroup.

The importance of somatic mutations and their impact on MDS diagnosis and outcomes derive from studies on risk classification. Several mutations have been incorporated in risk classification-based systems. Studies have shown that integrating mutational analysis into the IPSS-R it could help in assigning MDS patients into different prognostic groups [64].

Mutations in *TP53*, *RUNX1*, and *NRAS* are often found in patients with high bone marrow blasts. Except for *SF3B1* mutations which have been found to hold a favorable impact on survival, many other genes have been associated with poor prognosis. This is the case of the other two spliceosomal genes (*SRSF2*, *U2AF1*), epigenetic modifiers (*ASXL1*, *IDH1/2*, *DNMT3A*, *EZH2*) and signaling pathways (*CBL*, *NRAS*). Three mutations (*ASXL1*, *TP53*, *RUNX1*) were found independent predictors of higher rates of relapse and predicted for low survival outcomes after HSC transplantation in MDS and MDS/AML. Moreover, enhanced prediction of prognostic models have been achieved with the incorporation of a combination of mutations (*ASXL1*, *ETV6*, *EZH2*, *TP53*, *RUNX1*) [85]. Furthermore, with the discovery of additional gene mutations, studies have also reported that the total number of oncogenic mutations and cytogenetic lesions negatively associated with leukemia-free survival [101]. In Table 2, we summarize the clinical characteristics, prognoses, and potential targeted treatments in MDS patients with selected genetic abnormalities.

## 8. Molecular Targeted Therapies

MDS is characterized by the occurrence of somatic mutations in several cellular pathways as previously described. Hence, these pathways and their key genes represent proposed targets for pharmacologic therapy.

Therapeutic interventions for splicing factor mutations have been based on the development of pan-splicing modulators. Bacterially derived products and analogs have shown to bind the SF3B complex and disrupt spliceosome assembly. Those compounds include a class at low (FR901463, FR901464, FR901465, herboxidienes, pladienolides) and high stability [E7107 (an analog of pladienolide B), spliceostatin A (SSA; from FR901464), and the sudemycins]. Recently, H3B-8800, a selective and orally bioavailable modulator of wild type and mutant SF3b, showed a dose-dependent modulation of splicing. H3B-8800 is a small molecule that was designed on the scaffold of pladienolide with a potency of binding to SF3b complexes. Oral administration of H3B-8800 demonstrated preferential induction of apoptosis in several xenograft models bearing spliceosomal mutations. Data from the phase 1/2 study evaluating the pharmacokinetics/pharmacodynamics in MDS and related disorders (NCT02841540) were recently reported [151].

Luspatercept (ACE-536) is a TGF-α sequester and a suppressor of the SMAD2/3 levels that had improved response in low-grade MDS carrying *SF3B1* mutations by increasing erythroid maturation and hemoglobin levels. In a double-blind, placebo-controlled, phase 3 trial in patients with very-low-risk, low-risk, or intermediate-risk MDS, 93% of the treated patients harbored an *SF3B1* mutation [152].

HSC showed sensitivity to treatment with aryl sulfonamides (e.g., indisulam). In fact, drug sensitivity correlated with increased DCAF15 expression levels. Indisulam and other sulfonamides seem to induce the degradation of RBM39 (RNA binding motif protein 39), causing abnormal mRNA splicing changes such as intron retention and exon skipping [153]. Moreover, protein arginine methyltransferases (PRMTs) inhibitors (MS023, GSK591) influenced the growth of *SRSF2* mutant cells [143].

Early-phase clinical trials (NCT03433781, NCT03397173) are underway to test the effects of ascorbic acid on MDS patients with *TET2* deficiency in vivo. Vitamin C was reported to restore the hematopoiesis in mouse models and primary cells with *TET2* deficiency [154].

Targeting *IDH1/2* mutants using small-molecule inhibitors is another active area of investigation. Enasidenib (AG-221) is an oral inhibitor of *IDH2* mutant proteins that are involved in the citric acid cycle and catalyze the conversion of isocitrate to α-ketoglutarate (α-KG). When IDH2 mutations occur, mutant IDH2 proteins elicit neomorphic activity that reduces α-KG to the oncometabolite (R)-2-hydroxyglutarate (2-HG). Increased 2-HG levels inhibit α-KG proteins (histone demethylases, TET family-DNA methylcytosine dioxygenases) inducing hypermethylation of histones and arresting cellular differentiation. AG-221 treatment restores 2-HG levels to normal and unblocks the differentiation of *IDH2* mutant cells. Ivosidenib (AG-120) is a highly specific and reversible inhibitor of mutant *IDH1* [155]. AG-120 works through the allosteric competition with the magnesium ion, which prevents the generation of a catalytically active site.

Novel therapeutic agents, mostly activators, have been generated for mutant p53. PRIMA-1Met (APR-246, APR) is an investigational small molecule which restores the conformation of p53 and rescues p53 function [156]. More recently, it was reported that low doses of APR-246 alone or with 5-azacitidine, reactivate p53 and induce apoptosis in *TP53* mutant MDS and AML cell lines and primary cells [157].

Finally, allogeneic hematopoietic cell transplantation (allo-HCT) is a potentially curative treatment option; however, relapse occurs in 10–50% of patients with MDS after allo-HCT [158,159]. High-risk cytogenetics is associated with early relapse, and most relapses after allo-HCT are postulated to be due to regrowth of the pre-transplant MDS clones or emergence of new MDS clones [159]. Donor cell MDS and donor cell leukemia are a very rare and complex phenomenon and are only described in the literature as case reports and case series [160,161]. The underlying pathogenesis for donor cell MDS is unknown. Treatment options for these patients are limited and associated with poor prognosis [162]. Chromosome 7 monosomy is the most common karyotype abnormality reported in patients with donor cell MDS/DCL [163,164].

## 9. External Risk Factors of MDS: “Environmental Exposures”

The incidence of MDS is more common in males than females, except for isolated del(5q). There is no clear explanation for the difference in incidence; however, increased occupational/environmental exposure to toxins in the male may be the contributing factor. Environmental exposure to toxins such as arsenic, metals (copper, nickel, and steel), inorganic dust (asbestos and silica), is found to be associated with a cytogenetic abnormality in patients with MDS and AML. Exposure to inorganic gases and asbestos is found to be associated with chromosomal 5 and 7 abnormalities. On the other hand, exposure to organic toxins, metals, and radiation is associated with chromosomal 8 abnormalities [165]. Perhaps, these cytogenetic abnormalities can be possibly explained by the oxidative stress leading to DNA/cellular damage [166]. So far, only benzene is known to be strongly correlated with MDS and AML [167,168]. In vitro study demonstrated chromosomal damage with benzene metabolites in human lymphocytes [169]; however, individuals exposed to benzene had higher gene duplicating mutations in the glycophorin A mutational assay than gene-inactivating mutations [170]. Besides, cigarette smoking is another source of environmental exposure to benzene and is postulated to have a predisposition to MDS/AML. A high level of benzene was detected in smokers and was associated with an increased risk of AML in chronic benzene exposure [168,171]. A meta-analysis by Tong H et al. demonstrated a 45% increased risk of MDS in ever-smoker as compared to non-smoker [172]. The authors also reported a higher risk of MDS in women (ever-smokers), heavy smokers (≥20 cigarettes/day), and individuals with a history of smoking >20 pack-years. Nonetheless, the precise pathogenesis of smoking in AML/MDS is unknown.

## 10. Conclusions

MDS is a complex disease with a fascinating origin. A plethora of molecular pathway disruptions have been postulated to explain the heterogeneity of the disease phenotype, but no exclusive genetic drivers are capable of recapitulating all its aspects. The recent advances in next-generation sequencing were crucial and hallmarked specific gene mutations that are now part of disease risk stratification and targeted therapeutic modalities. This is the case of *SF3B1* mutations, as a part of the 2016 World Health Organization diagnostic criteria of MDS-RS and *TP53* mutations which identify a distinct disease entity of poor prognosis. Future advancements in the understanding of MDS molecular mechanisms will further enhance the guidance of clinical decisions to help move forward into individualized therapeutic approaches.

## Figures and Tables

**Table 1:** Clinical and molecular characteristics of ICUS, CHIP, and CCUS.

	Idiopathic Cytopenia of Undetermined Significance (ICUS)	Clonal Hematopoiesis of Indeterminate Potential (CHIP)	Clonal Cytopenia of Undetermined Significance (CCUS)
Incidence	NA/unknown	>10% in age ≥70 years	40% of patients with ICUS
Dysplasia	<10%	<10%	<10%
Cytopenia ^#^	Present	Absent	Present
Clonality	Absent	Present	Present
Bone marrow blast %	<5%	<5%	<5%
Mutation in the leukemia-associated gene	Absent	Present	Present
Variant allele frequency %	<2%	≥2%	≥2%
Common mutated somatic genes	*U2AF1*, *SRSF2*, *ASXL1*, *TP53*, *TET2*, *DNMT3A*	*DNMT3A*, *TET2, ASXL1*, *TP53*, *JAK2*	*TET2*, *DNMT3A*, *ASXL1*, *TP53*, *U2AF1*, *ZRSR2*, *SRSF2*, *JAK2*, *RUNX1*
Risk of progression to MDS	Low	Very low	High risk
Risk of progression to AML	Very low	Very low	Low
Rate of progression to MDS/AML	Variable	0.5–1% per year	Variable
Risk of cardiovascular disease	Unknown	Increased risk	Unknown
Outcome/Mortality ^$^	Unknown	Increased mortality	Unknown

Abbreviations: ICUS, idiopathic cytopenia of undetermined significance; CHIP, clonal hematopoiesis of indeterminate potential; CCUS, clonal cytopenia of undetermined significance NA, not available; MDS, myelodysplastic syndrome; AML, acute myeloid leukemia # Cytopenia includes unexplained anemia, thrombocytopenia, and neutropenia. Besides, it is imperative to rule out other etiology of a cytopenia such as dietary deficiencies, chronic liver disease, chronic kidney disease, idiopathic thrombocytopenia and neutropenia, autoimmune diseases, drug-induced cytopenia, and viral infections (Human immunodeficiency virus, Epstein–Barr virus, hepatitis virus). $ Overall survival is decreased in patients with CHIP as compared to the general population mainly due to the increased risk of cardiovascular disease.

**Table 2:** The clinical characteristics, prognoses, and potential targeted treatments in MDS patients with selected genetic abnormalities.

**Genetic Abnormality**	**Mutational Frequency**	**Clinical Characteristics**	**Therapeutics or Potential Targeted Therapy**	**Prognosis**	**Additional Comments**
del(5q)	10–15%	Anemia with or without neutropenia/thrombocytosis	Lenalidomide	Good	Isolated del(5q) is the only cytogenetic abnormality identified as a specific subtype in the WHO classification of MDS
del(7q)	50% (t-MDS), 10% (de novo MDS)	Severe cytopenias, increased risk of infection	Hypomethylating agents	Intermediate	
trisomy 8	5–10%	Cytopenias, autoimmune manifestations	Immunosuppressive agents	Intermediate	
del(20q)	2%	Thrombocytopenia	NA	Good	
del(17p)	1%	Associated with t-MDS	NA	Very poor	
-Y	NA	Benign clinical course	NA	Very good	Age-related phenomenon
-X	Rare	Usually benign course	NA	Intermediate	Age-related phenomenon
Complex karyotype with 3 chromosomal abnormalities	10%	Cytopenias, increased resistance to chemotherapeutic agents	NA	Poor	Complex karyotype with >3 chromosomal abnormalities confers very poor prognosis
*TP53* mutation	5–20%	Commonly found in t-MDS and AML	Hypomethylating agents, APR-246	Poor	*TP53* is an independent marker for prognosis and relapse even after allogeneic BMT
*TP53* mutation and del(5q)	20%	Aggressive disease with increased risk for transformation to AML	Hypomethylating agents	Poor	No response to lenalidomide
*TP53* mutation and complex karyotype		High bone marrow blast proportion, severe anemia, thrombocytopenia	Hypomethylating agents	Very poor	

Abbreviations: NA, not available; del, deletion, WHO, world health organization; t-MDS, therapy-related myelodysplastic syndrome; AML, acute myeloid leukemia.
